# Mild Moxibustion Decreases the Expression of Prokineticin 2 and Prokineticin Receptor 2 in the Colon and Spinal Cord of Rats with Irritable Bowel Syndrome

**DOI:** 10.1155/2014/807308

**Published:** 2014-06-12

**Authors:** Cili Zhou, Jimeng Zhao, Luyi Wu, Renjia Huang, Yin Shi, Xiaomei Wang, Wen Liao, Jue Hong, Shimin Liu, Huangan Wu

**Affiliations:** ^1^Key Laboratory of Acupuncture-Moxibustion and Immunological Effects, Shanghai University of Traditional Chinese Medicine, Shanghai 200030, China; ^2^Yueyang Hospital of Integrated Traditional Chinese and Western Medicine, Shanghai University of Traditional Chinese Medicine, Shanghai 200437, China; ^3^Center for Prescription and Syndrome of Traditional Chinese Medicine and Systems Biology, Shanghai University of Traditional Chinese Medicine, Shanghai 201203, China

## Abstract

It has been proven that prokineticin 2 (PK2) and its receptor PKR2 play an important role in hyperalgesia, while mild moxibustion can relieve visceral hypersensitivity in a rat model of irritable bowel syndrome (IBS). The goal of the present study was to determine the effects of mild moxibustion on the expression of PK2 and PKR2 in colon and spinal cord in IBS rat model, which was induced by colorectal distension using inflatable balloons. After mild moxibustion treatment, abdominal withdrawal reflex (AWR) scores were assessed by colorectal distension; protein and mRNA expression of PK2 and PKR2 in rat colon and spinal cord was determined by immunohistochemistry and fluorescence quantitative PCR. Compared with normal rats, the AWR scores of rats and the expressions of PK2/PKR2 proteins and mRNAs in colon and spinal cord tissue were significantly increased in the model group; compared with the model group, the AWR scores of rats and the expressions of PK2/PKR2 proteins and mRNAs in colon and spinal cord tissue were significantly decreased in the mild moxibustion group. These findings suggest that the analgesia effect of mild moxibustion may be associated with the reduction of the abnormally increased expression of the PK2/PKR2 proteins and mRNAs in the colon and spinal cord.

## 1. Introduction

Irritable bowel syndrome (IBS) is a functional bowel disorder characterized by abdominal pain or discomfort accompanied by changes in bowel habits [1–3]. The incidence of IBS in China is 5–20.72% [4, 5]. Clinical symptoms such as abdominal pain and diarrhea severely affect the quality of life for IBS patients. Studies have shown that visceral hypersensitivity and abnormal gastrointestinal motility are the main pathogenic factors that cause clinical symptoms such as abdominal pain and diarrhea in IBS patients [6–8]. Reducing visceral hypersensitivity and improving gastrointestinal mobility are the major strategies to improve quality of life for IBS patients.

Prokineticins (PKs) are newly identified peptide family members in mammals. The PK family mainly includes PK1 and PK2. PK1 is the human homolog of a nontoxic protein named venom protein A (VPRA) or mamba intestinal toxin 1 (MIT1) isolated from black mamba venom. PK2 is the human homolog of the protein* Bombina variegate* (Bv8) isolated from skin secretions of the toad. Through G protein-coupled receptors termed prokineticin receptors (PKRs), PKs participate in a variety of biological processes in the body, including regulation of gastrointestinal mobility and transmission of pain signals [9–14]. PKs were initially discovered as regulators of bowel function and are a new type of endogenous regulator of gastrointestinal motility that specifically lead to gastrointestinal smooth muscle contraction, as evidenced by the observation that they specifically induce the contraction of the ileum longitudinal muscle, the basal muscle, and the proximal colon in guinea pigs [15, 16]. Subsequent studies showed that PKs mediate pain signal transmission and sensitize nociceptors. Through spinal cord and primary sensitive neurons, PKs strongly sensitize peripheral nociceptors to thermal, chemical, and mechanical stimuli and are directly involved in nociceptive threshold changes and nociceptive responses caused by noxious stimuli, thus mediating primary and central pain sensitization. Therefore, inhibition or antagonism of PKs/PKRs can relieve pain [17–21].

Currently, the mechanism of pathogenesis of IBS is not fully understood. Investigation into the association between PK/PKR expression and IBS could further elucidate the mechanism of IBS and provide new targets for IBS treatment. Our group established a rat IBS model based on colorectal distension in neonatal rats stimulated by inflatable balloons according to Al-Chaer et al. [22]. Our previous studies showed that (1), compared with normal rats, pain threshold dropped and visceral sensitivity increased as well as PK1 and PKR1 expression significantly enhanced in the colon and spinal cord in IBS model rats; (2) mild moxibustion was effective at treating IBS as it increased the pain threshold, improved visceral hypersensitivity, and decreased PK1 and PKR1 expression in the colon and spinal cord in IBS model rats [23, 24]. These indicated that PKs and PKRs are closely associated with IBS; PK1 and PKR1 are involved in pathophysiology of IBS and mild moxibustion analgesia in IBS.

PKs and PKRs mainly include PK1, PKR1, PK2, and PKR2. Thus far, reports on the association between PK2/PKR2 and IBS are scarce. Based on previous studies, this study further investigated the mechanism of IBS and mild moxibustion in treating IBS from the perspective of PK2/PKR2.

## 2. Materials and Methods

### 2.1. Experimental Animals

A total of 42 male neonatal rats (5-day-old) were provided by the Department of Laboratory Animal Science at Shanghai Medical College of Fudan University. The animals were maintained under light-dark conditions (12 h light : 12 h dark) at a room temperature of 20 ± 2°C with a relative humidity of 50–70%. This study was performed in accordance with the National Institutes of Health Guide for the Care and Use of Laboratory Animals.

After three days of habituation, the neonatal rats were randomly divided into 3 groups: the normal group, the model group (only modeling), and mild moxibustion group (modeling followed by mild moxibustion).

### 2.2. Establishment of the IBS Model

The chronic visceral hypersensitivity rat model was established according to Al-Chaer et al. [22].

An inflatable balloon was slowly inserted to a depth of 2 cm through the anus along the colorectal physiological curvature. The balloon was distended with 0.2 mL of air for 1 min and was then deflated and withdrawn slowly. The same stimulation procedure was repeated after 30 min. The balloon stimulation was performed twice a day, for 14 days.

### 2.3. Mild Moxibustion Treatment

After the model was established, rats in the mild moxibustion group began to receive mild moxibustion treatment at 7th week. The diameter of moxa sticks (Nanyang Hanyi Moxa Co., Ltd., Nanyang, Henan province, China) was 0.5 cm. When the moxa sticks were ignited, moxibustion was performed at 2 cm above bilateral Tianshu points (ST 25, 5 cun above the pubic symphysis and 2 cun away from the midline) simultaneously for 10 min. Moxibustion was conducted once a day for a total of 7 days.

### 2.4. Abdominal Withdrawal Reflex (AWR) Scoring

According to the method of Al-Chaer et al. [22], AWR scoring was conducted on rats within 90 min after 7 moxibustion treatments. Balloons were slowly inserted into the descending colons through the anus, and four intensities, 20, 40, 60, and 80 mmHg, of colorectal distention (CRD) were applied. Each CRD lasted 20 s every 4 minutes and repeated 5 times. AWR score was assessed by two researchers. The data for each animal were averaged for analysis.

The criteria for AWR scoring [22] were as follows: 0, no behavioral response to CRD; 1, brief head movement followed by immobility during CRD; 2, mild contraction of the abdominal muscles but no lifting; 3, strong contraction of the abdominal muscles and lifting of the abdomen without lifting the pelvic structure and scrotum; and 4, body arching and lifting of the pelvic structure and scrotum.

### 2.5. Preparation of Colon and Spinal Cord Tissue Samples

After AWR scoring, rats were weighed and anesthetized using 3% sodium pentobarbital (0.1 mL/100 g). Six rats in each group were sacrificed, and their colon (3 cm in length, 5 cm above the anus) and spinal cord tissue (at the lumbar enlargement) were collected immediately. The samples were stored in a −80°C freezer for subsequent fluorescence-based quantitative PCR (FQ-PCR) detection. The remaining rats in each group received a rapid left-ventricular perfusion with 250 mL saline until the liver became white, followed by perfusion and fixation with 500 mL 4% paraformaldehyde. After perfusion, 3 cm of colon tissue located 5 cm above the anus and spinal cord tissue at the lumbar enlargement was removed and fixed in 4% paraformaldehyde (for less than 24 h) for subsequent immunohistochemical detection.

### 2.6. Immunohistochemical Assay for Expression of PK2 and PKR2

Paraffin sections of colon and spinal cord tissue were deparaffinized to water. After incubation with 3% H_2_O_2_ at room temperature for 20 min, sections were immersed in 0.01 M citrate buffer (pH 6.0) and heated twice in a microwave oven with medium heat until boiling. Then sections were blocked using 5% bovine serum albumin (BSA) at room temperature for 20 min, and the primary antibodies were added dropwise (PK2: rabbit anti-rat polyclonal antibody, 1 : 400, Novus Co., Littleton, USA; PKR2: rabbit anti-rat polyclonal antibody, 1 : 100, Wuhan USCN Life Co., Wuhan, China) in a moisture chamber at 4°C overnight followed by incubation in a 37°C incubator for 2 h. Corresponding secondary antibody (biotinylated goat anti-rabbit IgG, 1 : 100, Wuhan Boster Bio-Engineering Co., Ltd., Wuhan, China) was then added dropwise, and sections were incubated at 37°C for 20 min. After incubation with streptavidin-biotin complex (SABC) (Wuhan Boster Bio-Engineering Co., Ltd., Wuhan, China) at 37°C for 20 min, sections were developed using 3,3′-diaminobenzidine (DAB) (Wuhan Boster Bio-Engineering Co., Ltd., Wuhan, China). After counterstaining with hematoxylin for 1 min, sections were dehydrated, cleared, mounted, and observed under a light microscope (BH2, Olympus, Tokyo, Japan).

Positive signals were represented by a brown-yellow color. Image analysis was performed using the Motic Med 6.0 digital medical image analysis system (Motic Group Co., Ltd., Xiamen, China). Three nonoverlapping fields of each sample were randomly selected, and the data of the integral optical densities of positive signals for each sample were average for analysis. During image analysis process, the intensity of the light source was the same for all samples.

### 2.7. FQ-PCR Assay for Expression of PK2 mRNA and PKR2 mRNA

Total RNA in colon and spinal cord tissue was extracted using Trizol and reversely transcribed into cDNA according to the manufacturer's instruction manual. The reaction system consisted of 4 *μ*L of 5x reverse transcription buffer, 0.5 *μ*L of oligo (dT), 0.5 *μ*L of dNTPs, 1 *μ*L of MMLV reverse transcriptase, 10 *μ*L of DEPC-treated water, and 4 *μ*L of RNA template; the total volume was 20 *μ*L. The reaction conditions were 37°C for 1 h followed by 95°C for 5 min to inactivate the MMLV reverse transcriptase. The prepared cDNA was used for PCR amplification. The amplification system consisted of 10 *μ*L of 5x PCR buffer, 0.5 *μ*L of upstream primer (F), 0.5 *μ*L of downstream primer (R), 0.5 *μ*L of dNTPs, 0.5 *μ*L of TaqMan fluorescent probe, 1 *μ*L of Taq polymerase, 32 *μ*L of ddH_2_O, and 5 *μ*L of cDNA template; the total volume was 50 *μ*L. The amplification process included 40 cycles of 50°C for 2 min, 95°C for 5 min, 95°C for 15 s, and 60°C for 45 s.

The synthesis and purification of probes and primers were provided by Da'an Bio-Technology Co,. Ltd., Qingdao, China. GADPH was used as an internal control. The sequence of the GADPH probe was 5′-CATCTGGGCTACACTAGGACCA-3′; the sequence of the upstream primer for GADPH was 5′-GCTGTTGAGTCACAGGAGCAA-3′, and the downstream primer was 5′-CCGAGGGCCCACTAAAGG-3′. The sequence of the PK2 probe was 5′-TTGCTGCTACCGCTGCTGCTCACAC-3′; the sequence of the upstream primer for PK2 was 5′-CCTCCACACTGAGAGTCCTTG-3′, and the downstream primer was 5′-GTGCCCCGCTACTGCTAC-3′. The sequence of the PKR2 probe was 5′-TGTGCCTCCGTCAACTACCTTCGT-3′, the sequence of the upstream primer for PKR2 was 5′-GAGGCGGTCTGGTAATTCATCC-3′, and the downstream primer was 5′-CTTTCCTGGGAGCATGGTCAC-3′.

The mRNA expressions of the target genes were analyzed using the ABI Prism 7300 SDS software. The relative mRNA expression of the target gene = 2^−ΔCT^ × 100%, where ΔCT = CT value of the target gene-CT value of the internal control (GADPH).

### 2.8. Statistical Analysis

Statistical analysis was performed using the SPSS18.0 software. Statistical description: data are presented as mean ± SD for normal distribution and as M, min-max, for nonnormal distribution. Statistical inference: one-way analysis of variance (one-way ANOVA) was performed if the data followed a normal distribution; the nonparametric test was performed if the data did not follow a normal distribution. If variances were homogeneous, the difference between groups was also compared using the least significant difference (LSD) test; if variances were not homogeneous, the difference between groups was compared using the Games-Howell test. *P* < 0.05 was considered statistically significant.

## 3. Results

### 3.1. Quantitative Analysis of Experimental Animals

In total, 40 rats were included in the statistical analysis. One rat in the normal group was lost during perfusion and fixation, and one rat of the model group died after anesthesia.

### 3.2. The Analgesic Effects of Mild Moxibustion on Chronic Visceral Hypersensitivity in IBS Model Rats

**Figure 1:**
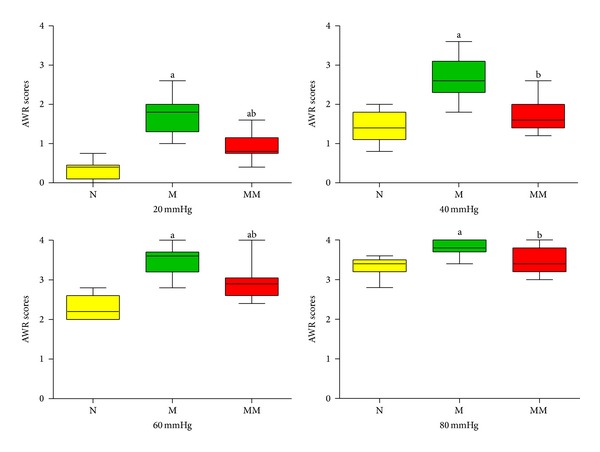
AWR scores of rats in each group. Data are presented as M, min-max. Under the same intensity of CRD stimulation, compared with the normal group: ^a^ *P* < 0.01; compared with the model group: ^b^ *P* < 0.01. N: normal group; M: model group; MM: mild moxibustion group.

The AWR scores of rats in each group after different intensities (20 mmHg, 40 mmHg, 60 mmHg, and 80 mmHg) of CRD stimuli are shown in Figure 1. Compared with the normal group, the AWR scores at all intensities (20 mmHg, 40 mmHg, 60 mmHg, and 80 mmHg) of rats in the model group significantly increased, with *P* < 0.01. Compared with the model group, the AWR scores of rats at all intensities (20 mmHg, 40 mmHg, 60 mmHg, and 80 mmHg) in the mild moxibustion group significantly decreased, with *P* < 0.01.

### 3.3. The Effects of Mild Moxibustion on the Expression of PK2 and PKR2 in the Colons of Chronic Visceral Hypersensitivity Model Rats

**Figure 2:**
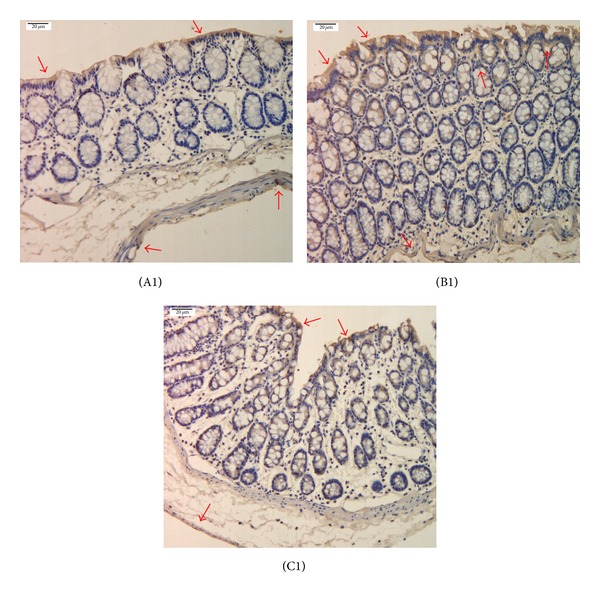
PK2 expression in the colon of chronic visceral hypersensitivity model rats (immunohistochemical detection). Yellow stains denote PK2-positive expression as shown by the arrows. PK2 was mainly expressed in the mucosal epithelium, the proximal glands of the mucosal epithelium, the interstitium, and the muscle layer of the colon. (A1) normal group, (B1) model group, and (C1) mild moxibustion group. Scale bar: 20 *μ*m.

**Figure 3:**
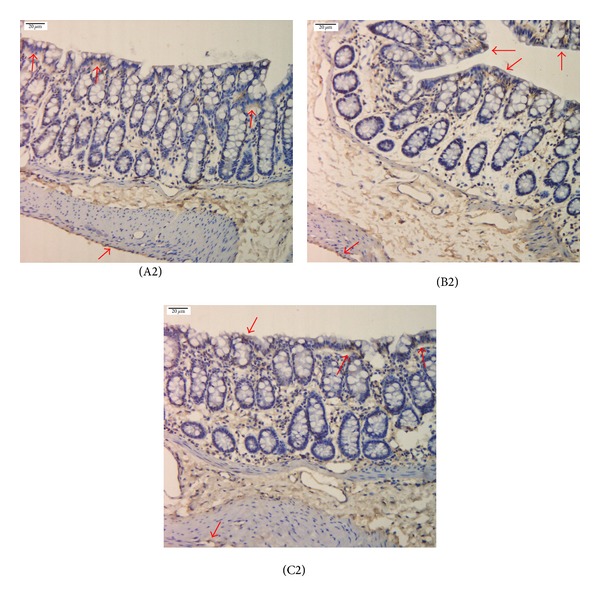
PKR2 expression in the colon of chronic visceral hypersensitivity model rats (immunohistochemical detection). Yellow stains denote PKR2-positive expression as shown by the arrows. PKR2 was mainly expressed in the mucosal epithelium, the proximal glands of the mucosal epithelium, the interstitium, and the muscle layer of the colon. (A2) normal group, (B2) model group, and (C2) mild moxibustion group. Scale bar: 20 *μ*m.

**Figure 4:**
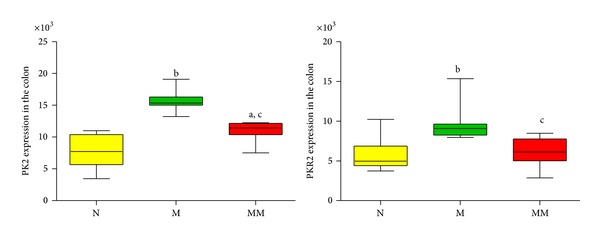
The integral optic density of the expression of PK2 and PKR2 in the colon of each group. Data are presented as M, min-max. Compared with the normal group: ^a^ *P* < 0.05, ^b^ *P* < 0.01; compared with the model group: ^c^ *P* < 0.01. N: normal group; M: model group; MM: mild moxibustion group.

Immunohistochemistry showed that compared with the normal group, the expression of PK2 and PKR2 in the colons of the model group significantly increased, with *P* < 0.01. After mild moxibustion treatment, compared with the model group, the expression of PK2 and PKR2 in the colon significantly decreased, with *P* < 0.01 (Figures 2, 3, and 4).

### 3.4. The Effects of Mild Moxibustion on the Expression of PK2 and PKR2 in the Spinal Cord of Chronic Visceral Hypersensitivity Model Rats

**Figure 5:**
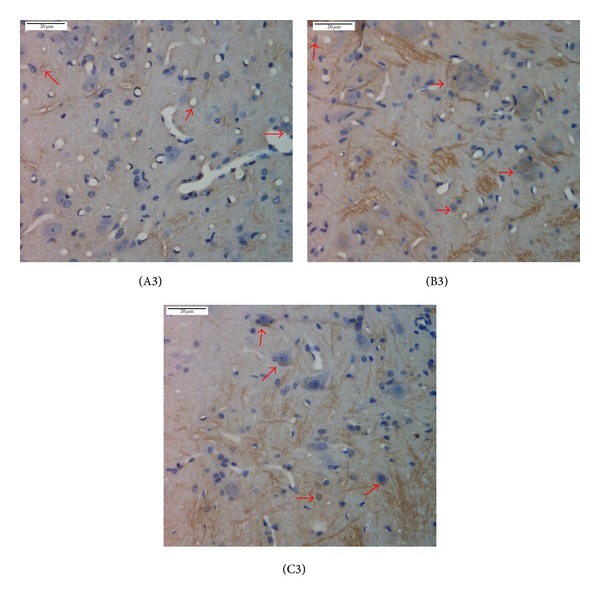
PK2 expression in the spinal cord of chronic visceral hypersensitivity model rats (immunohistochemical detection). Yellow stains denote PK2-positive expression as shown by the arrows. PK2 was mainly expressed in the large, medium, and small neurons, vascular endothelial cells, and the interstitium of the spinal cord. (A3) normal group, (B3) model group, and (C3) mild moxibustion group. Scale bar: 20 *μ*m.

**Figure 6:**
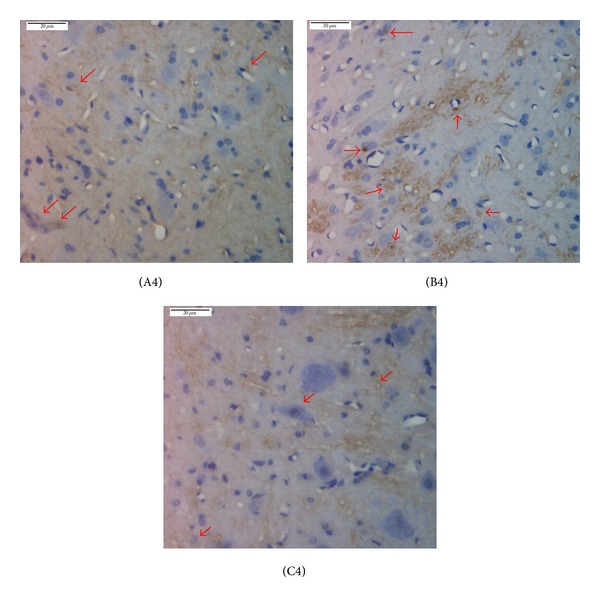
PKR2 expression in the spinal cord of chronic visceral hypersensitivity model rats (immunohistochemical detection). Yellow stains indicate PKR2-positive expression as shown by the arrows. PKR2 was mainly expressed in the large, medium, and small neurons, vascular endothelial cells, and the interstitium of the spinal cord. (A4) normal group, (B4) model group, and (C4) mild moxibustion group. Scale bar: 20 *μ*m.

**Figure 7:**
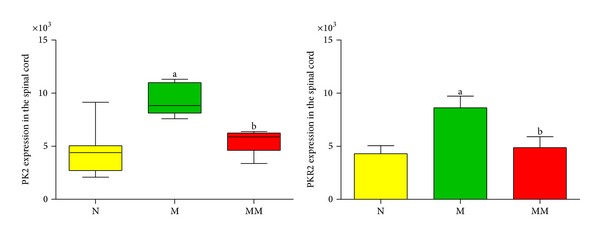
The integral optic density of the expression of PK2- and PKR2-positive signal in the spinal cord of each group. Data of PK2 expression are presented as M, min-max, whereas the data of PKR2 expression are presented as mean ± SD. Compared with the normal group: ^a^ *P* < 0.01; compared with the model group: ^b^ *P* < 0.01. N: normal group; M: model group; MM: mild moxibustion group.

Immunohistochemistry showed that, compared with the normal group, the expression of PK2 and PKR2 in rat spinal cord in the model group significantly increased, with *P* < 0.01. After mild moxibustion treatment, compared with the model group, the expression of PK2 and PKR2 significantly decreased, with *P* < 0.01 (Figures 5, 6, and 7).

### 3.5. The Effects of Mild Moxibustion on the Expression of PK2 and PKR2 mRNA in the Colon of Chronic Visceral Hypersensitivity Model Rats

**Figure 8:**
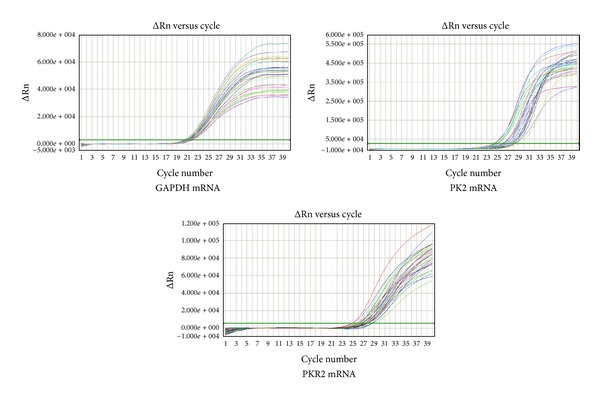
The amplification kinetics curve of PK2 mRNA, PKR2 mRNA, and GAPDH mRNA in the colon in each group.

**Figure 9:**
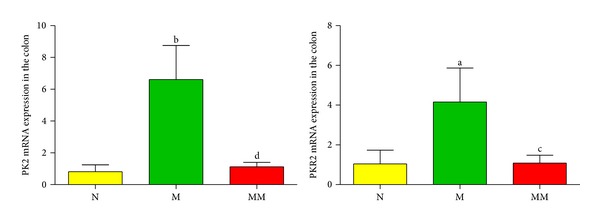
The relative expression levels of PK2 and PKR2 mRNA in the colon in each group (%). Data are presented as mean ± SD. Compared with the normal group: ^a^ *P* < 0.05, ^b^ *P* < 0.01; compared with the model group: ^c^ *P* < 0.05, ^d^ *P* < 0.01. N: normal group; M: model group; MM: mild moxibustion group.

The amplification kinetics curves of PK2 mRNA, PKR2 mRNA, and GAPDH mRNA (internal control) in the colon in each group are shown in Figure 8.The results of FQ-PCR demonstrated that, compared with the normal group, the expression of PK2 and PKR2 mRNA in the colon in the model group was significantly increased (*P* < 0.05, *P* < 0.01). After mild moxibustion treatment, the expression of PK2 and PKR2 mRNA in the colon significantly decreased compared with the model group (*P* < 0.05, *P* < 0.01) (Figure 9).

### 3.6. The Effects of Mild Moxibustion on the Expression of PK2 and PKR2 mRNA in the Spinal Cord of Chronic Visceral Hypersensitivity Model Rats

**Figure 10:**
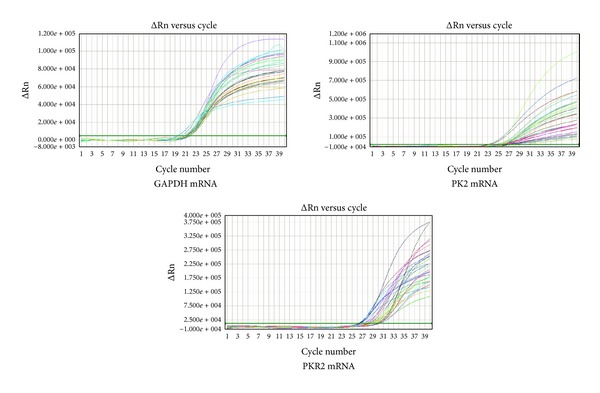
The amplification kinetics curve of PK2 mRNA, PKR2 mRNA, and GAPDH mRNA in the spinal cord in each group.

**Figure 11:**
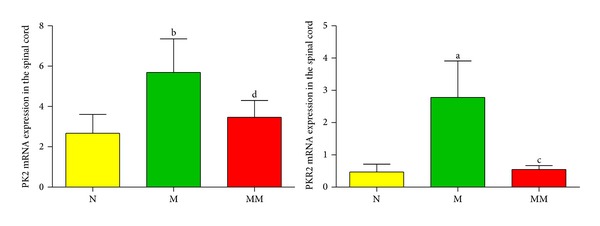
The relative expression levels of PK2 and PKR2 mRNA in the spinal cord in each group (%). Data are presented as mean ± SD. Compared with the normal group: ^a^ *P* < 0.05, ^b^ *P* < 0.01; compared with the model group: ^c^ *P* < 0.05, ^d^ *P* < 0.01. N: normal group; M: model group; MM: mild moxibustion group.

The amplification kinetics curves of PK2 mRNA, PKR2 mRNA, and GAPDH mRNA (internal control) in the spinal cord in each group are shown in Figure 10. The results of FQ-PCR showed that the expression of PK2 and PKR2 mRNA in the spinal cord in the model group significantly increased compared with the normal group (*P* < 0.05, *P* < 0.01). After mild moxibustion treatment, the expression of PK2 and PKR2 mRNA in spinal cord tissue significantly decreased compared with the model group (*P* < 0.05, *P* < 0.01) (Figure 11).

## 4. Discussion

Previous studies showed that PKs and PKRs participate in the sensitization of nociceptors and hyperalgesia and are closely associated with the transmission of pain signals. Injection of a small amount of PK/Bv8 into the central nervous system [16, 25] and peripheral nervous system [26] of rats produced strong hyperalgesia in a dose-dependent manner [24–27]. A study by Negri et al. [17] showed that intravenous, subcutaneous, and intrathecal injection of Bv8 (the frog homolog of PK2) systemically induced an intense sensitization of nociceptors to mechanical and thermal stimuli in rats. Lacking PKs/PKRs significantly decreased sensitivity to noxious stimuli and reduced pain sensation in rodents [28–30]. Hu et al. [28] reported that mice lacking the PK2 gene displayed a reduction in nociception induced by thermal and chemical stimuli including capsaicin and exhibited significantly reduced late-phase responses to subcutaneous administration of formalin. In acute and chronic inflammation models, mice with PKR-null had reduced pain behavior and significantly decreased inflammatory hyperalgesia [30, 31]. PKR antagonists reduced and eventually abolished PK1 and PK2-induced hypernociception and inflammatory hyperalgesia in a dose-dependent manner [31], indicating that PK2 and PKR2 play important roles in nociceptor sensitization and hyperalgesia, and mediate peripheral and central pain sensitization.

Abdominal pain is one of the major symptoms in IBS patients. Compared with normal individuals, IBS patients have increased pain sensitivity and reduced pain threshold [32–34]. When the balloon pressure in the rectum reached 40 mmHg, 90.7% of IBS patients had abdominal pain, and the specificity was 80% [35]. The severity of IBS symptoms is associated with pain threshold, and there is no significant difference among subtypes [36]. Therefore, increasing the pain threshold and relieving pain are effective methods for treating IBS. In this study, at different intensities of stimulation (20 mmHg, 40 mmHg, 60 mmHg, and 80 mmHg), the AWR scores of IBS model rats all significantly increased and were significantly different from those of the control group with *P* < 0.01. These results indicated that visceral pain sensitivity intensified and pain threshold decreased in rats after the establishment of the model. A study by Watson et al. [37] showed that PK2 gene expression in the gastrointestinal tract was significantly upregulated in ulcerative colitis patients and rodent models of colitis visceral pain induced by mustard oil, trinitrobenzene sulfonate, water-avoidance stress, and* Citrobacter rodentium* infection. Therefore, it was believed that enhanced PK2 expression induced visceral pain through the PKR2 pathway. In this study, IBS model rats had enhanced visceral pain sensitivity and reduced pain threshold; compared with the normal group, their PK2/PKR2 protein and mRNA expression in colon tissue were significantly increased (*P* < 0.05, *P* < 0.01), indicating that PK2 and PKR2 are associated with enhanced visceral pain sensitivity in IBS model rats.

In this study, immunohistochemistry showed that PK2 and PKR2 were mainly expressed in the mucosal epithelium, the proximal glands in the mucosal epithelium, the interstitium, and the muscular layer of the colon. The colon is innervated by five different types of afferent nerve fibers: serosal, mesenteric, muscular, mucosal, and muscular/mucosal. Different sensory and motor nerve impulses in the colon are transmitted from these afferent nerve fibers to the central nervous system through the dorsal root ganglion (DRG) [38]. The spinal cord is an essential pathway of sensory and motor nerve impulse conduction. Nerve impulses transmitted into the DRG are transmitted into the brain through the spinal cord. Physiological studies on somatic and visceral sensation nerves revealed that hyperexcitability of neurons in the dorsal horn, which can develop either in response to peripheral tissue irritation or in response to descending influences originating in the brainstem, plays a core role in chronic hyperalgesia in the gastrointestinal tract of the human body [39]. Coffin et al. [40] confirmed that IBS patients have spinal cord hyperexcitability through a study on the nociceptive flexion reflex in IBS patients. In this study, the expression of PK2/PKR2 protein and mRNA in the spinal cord of IBS model rats significantly increased compared with that of the normal group (*P* < 0.05, *P* < 0.01), indicating that PK2 and PKR2 also participate in the central mechanisms of visceral pain in IBS model rats.

Previous studies of our project team showed that mild moxibustion was an effective treatment for IBS [23, 24, 41]. In this study, after mild moxibustion treatment, the abnormally increased AWR scores at different intensities of stimuli (20 mmHg, 40 mmHg, 60 mmHg, and 80 mmHg) in IBS model rats all significantly decreased with *P* < 0.01. The results indicated that mild moxibustion effectively improved visceral hypersensitivity and increased pain threshold in rats with IBS, consistent with previous results [23, 24, 41]. Compared with the model group, the expression of PK2/PKR2 protein and mRNA in the colon and spinal cord of IBS model rats significantly decreased (*P* < 0.05, *P* < 0.01), indicating that mild moxibustion suppressed the abnormally increased expression of PK2/PKR2 protein and mRNA in the colon and spinal cord of IBS model rats. These findings suggest that PK2 and PKR2 are involved in mild moxibustion analgesia in rats with chronic visceral hyperalgesia.
